# Testing the Webber’s Comprehensive Mobility Framework Using Self-Reported and Performance-Based Mobility Outcomes Among Community-Dwelling Older Adults in Nigeria

**DOI:** 10.1093/geroni/igad019

**Published:** 2023-03-01

**Authors:** Ernest C Nwachuwku, Daniel Rayner, Michael C Ibekaku, Ekezie C Uduonu, Charles I Ezema, Michael E Kalu

**Affiliations:** Emerging Researchers & Professionals in Aging- African Network, Nigeria and Canada; Faculty of Health Science, McMaster University, Hamilton, Ontario, Canada; Emerging Researchers & Professionals in Aging- African Network, Nigeria and Canada; School of Physiotherapy, Dalhousie University, Halifax, Nova Scotia, Canada; Emerging Researchers & Professionals in Aging- African Network, Nigeria and Canada; Department of Medical Rehabilitation, Faculty of Health Science and Technology, University of Nigeria, Nsukka, Enugu State, Nigeria; Department of Medical Rehabilitation, Faculty of Health Science and Technology, University of Nigeria, Nsukka, Enugu State, Nigeria; Emerging Researchers & Professionals in Aging- African Network, Nigeria and Canada; School of Physiotherapy, Dalhousie University, Halifax, Nova Scotia, Canada

**Keywords:** Africa, Aging, Developing nation, Movement

## Abstract

**Background and Objectives:**

In 2010, Webber and colleagues conceptualized the interrelationships between mobility determinants, and researchers tested Webber’s framework using data from developed countries. No studies have tested this model using data from developing nations (e.g., Nigeria). This study aimed to simultaneously explore the cognitive, environmental, financial, personal, physical, psychological, and social influences and their interaction effects on the mobility outcomes among community-dwelling older adults in Nigeria.

**Research Design and Methods:**

This cross-sectional study recruited 227 older adults (mean age [standard deviation] = 66.6 [6.8] years). Performance-based mobility outcomes included gait speed, balance, and lower extremity strength, and were assessed using the Short Physical Performance Battery, whereas the self-reported mobility outcomes included inability to walk 0.5 km, 2 km, or climb a flight of stairs, assessed using the Manty Preclinical Mobility Limitation Scale. Regression analysis was used to determine the predictors of mobility outcomes.

**Results:**

The number of comorbidities (physical factor) negatively predicted all mobility outcomes, except the lower extremity strength. Age (personal factor) negatively predicted gait speed (β = −0.192), balance (β = −0.515), and lower extremity strength (β = −0.225), and a history of no exercise (physical factor) positively predicted inability to walk 0.5 km (*B* = 1.401), 2 km (*B* = 1.295). Interactions between determinants improved the model, explaining the most variations in all the mobility outcomes. Living arrangement is the only factor that consistently interacted with other variables to improve the regression model for all mobility outcomes, except balance and self-reported inability to walk 2 km.

**Discussion and Implications:**

Interactions between determinants explain the most variations in all mobility outcomes, highlighting the complexity of mobility. This finding highlighted that factors predicting self-reported and performance-based mobility outcomes might differ, but this should be confirmed with a large data set.

**Translational significance:** Mobility determinants’ (cognitive, environmental, financial, personal, physical, psychological, social) interrelatedness has been conceptualized and tested chiefly using data from developed regions (e.g., United States, Canada). Using data from developing regions (e.g., Nigeria) would provide a different perspective, as cultural practice in care provision for older adults differs from developed nations. Living arrangement was the only factor that consistently interacted with other determinants to improve the model in mobility outcomes. Amidst competing resources in developing regions of the world, interventions to increase social factors (e.g., social network or living arrangements) are promising to provide additional effects on other mobility determinants, enhancing mobility.

The world’s population is aging. Globally, the number of adults aged 60 and older is expected to double by 2050, rising from 962 million in 2017 to 2.1 billion in 2050 (United Nations, 2017). A substantial proportion of this global increase is expected to occur in underdeveloped regions, including Asia (65%), Africa (14%), and Latin America (11%; United Nations, 2019). Among this increase in older persons in Africa, Nigerian older adults are expected to make up a considerable proportion due to the country’s large population (World Population Review, 2021). Approximately 5% of the 200 million persons in Nigeria are aged 60 years and above, and this is projected to increase to 25.3 million individuals by 2050 (African Academy of Sciences, 2020). Increasing age has been associated with worsening health, greater chronic conditions, and decreasing functional capacity among older adults, all of which are associated with greater demands on the health care system (Beard et al., 2016; Suzman et al., 2015). The changes in the functional capacity that accompany aging are a substantial public health issue, and this has led to an increased focus on the determinants of mobility among older adults (World Health Organization, 2002).

In 2010, Webber et al. introduced a conceptual framework to identify potential mobility determinants, including physical, cognitive, social, psychological, environmental, and personal variables.^.^ The authors defined mobility as the ability to move oneself within a variety of environments, ranging from one’s home to their service community and beyond, and this movement can be done independently, with the use of an assistive device, or using transportation (e.g., driving, public services; Webber et al., 2010). Individual determinants within the model, such as low self-efficacy or depression (Gayman et al., 2008; Perkins et al., 2008), contribute to the restriction of mobility; however, the framework also proposes that these determinants are interrelated. For example, loss of vision or hearing (a physical factor) may elevate an individual’s anxiety regarding navigating challenging environments (e.g., crossing busy intersections), causing them to self-restrict their mobility. Likewise, financial factors, such as low personal income, may influence an individual’s access to public transport, ability to maintain social ties, and access to recreational facilities, all of which may limit their mobility. It is evident that as these determinants interact, their influence on mobility becomes more complex.

Several studies have applied Webber et al.’s framework to identify potential mobility determinants (Dunlap et al., 2021; Giannouli et al., 2019; Jafari et al., 2020; Jansen et al., 2017; Kuspinar et al., 2020; Ullrich et al., 2019; Umstattd Meyer et al., 2014). Ullrich et al. (2019) cross-sectionally explored determinants of life-space mobility among 118 cognitively impaired community-dwelling older adults in Germany and reported that motor performance, gender, and social and physical activity were independent predictors of mobility. Likewise, in a cross-sectional study, Giannouli et al. (2019) found that physical and psychological factors account for a small but significant portion of the variance in real-life mobility of 154 community-dwelling older Germans. More recently, using the Canadian Longitudinal Study on Aging (*n* = 12,646), Kuspinar et al. (2020) identified driving status, social support, and gait speed as the main correlates of life-space mobility among older adults.

Although these studies have focused mainly on life-space mobility, two studies have used structural equation modeling to test Webber et al.’s framework on other forms of mobility, including personal and community mobility (Umstattd Meyer et al., 2014) and mobility limitation (Jafari et al., 2020). Umstattd Meyer et al. (2014) explored mobility determinants among 6,112 U.S. older adults participating in the Health and Retirement Study and found that psychosocial, physical, environmental, and cognitive factors, as well as age and marital status, predicted self-reported personal and community mobility. A 2020 study recruited 1,201 Iranian older adults and reported that all determinants were significant predictors of mobility limitations in the expected direction; for instance, reduced physical activity level was associated with higher odds of reporting mobility limitations among Iranian older adults (Jafari et al., 2020).

Although these studies have highlighted the potential determinants of mobility among older adults, they present a few limitations. To the best of our knowledge, no study has explored Webber et al.’s framework in both self-reported and performance-based mobility within a single population. Performance-based and self-reported mobility measures have their advantages and limitations. Performance-based measures evaluate mobility in a controlled environment (Nielsen et al., 2016). Meanwhile, self-reported measures aim to capture the patient’s perspective on mobility and are often less costly and time consuming. However, these measures can be subject to bias through cognitive impairment or affective responses to illnesses (Nielsen et al., 2016). Overall, performance-based and self-reported assessments capture different forms of mobility, and past studies have noted discrepancies between the two measures (Bean et al., 2011; Roedersheimer et al., 2016; Sager et al., 1992). It is crucial to apply both performance-based and self-reported assessments to capture a comprehensive measure of mobility among older adults and highlight how factors influence each.

In addition, these studies that have explored Webber et al.’s framework among community-dwelling older adults have been predominantly restricted to high-income countries, including the United States, Canada, and Germany (Giannouli et al.., 2019; Kuspinar et al., 2020; Ullrich et al., 2019; Umstattd Meyer et al., 2014) or Iran—an upper-middle-income country (Jafari et al., 2020). Consequently, no studies have explored the determinants of mobility in developing nations, such as Nigeria. The United Nations’ World Population Ageing projections noted that developing countries would be facing pronounced demographic changes in the following decades (United Nations Department of Economic and Social Affairs, Population Division, 2020), making it crucial to identify the determinants of mobility relevant to these regions. Although the factors influencing older adults’ mobility could be similar across regions, there are significant differences in the cultural, environmental, demographic, and sociopolitical factors that affect health care systems differently. With no universal health coverage, older adults pay for their health care services and other services that aid mobility, such as transportation system, at no subsidized rate as in Western countries like the United States, Canada, and Germany (Saka et al., 2019). As well, many cities in Nigeria have poor environmental features (e.g., absence of walkways or crossings for pedestrians, high traffic congestion) and transportation systems (e.g., limited bus stops) compared to those in Western countries (Olawole & Aloba, 2014). It is plausible that these different environmental conditions and health care systems could limit the application of findings from studies conducted in Western countries. The purpose of this study is twofold: (a) to simultaneously explore the influences of physical, financial, cognitive, social, psychological, environmental, and personal factors on the performance-based (Short Physical Performance Battery [SPPB]) and self-reported (Manty Preclinical Mobility Limitation Scale) mobility outcomes among community-dwelling older adults in Nigeria, and (b) to investigate exploratively the interaction effects of the predictors on self-reported and performance-based mobility outcomes.

## Method

### Study Design

This cross-sectional study was conducted in two waves—Wave 1: April 2019 to June 2019 and Wave 2: November 2020 to January 2021. All participants underwent the same performance-based and self-reported mobility assessments. The Health Research Ethics Committee, University of Nigeria Teaching Hopsutal, Ituku Ozala- NHREC/05/01/2008B-FWA00002458-1RB000002323.

### Sampling and Sample Size

Multistage cluster sampling was employed to identify communities, and participants in the selected communities were conveniently sampled. All the local governments in the Enugu State of Nigeria were listed and numbered. Governments assigned even numbers that were systematically selected. Within the selected local governments, communities were systematically selected using the same process. Participants residing in the selected communities were invited to an organized health outreach, and they were conveniently sampled.

We estimated that a sample size of 164–172 is sufficient to have 80% power for the overall regression equation (i.e., adjusted *R*-squared of 0.142 [Giannouli et al., 2019] and 0.1350 [Kuspinar et al., 2020]) in our study, assuming a confidence interval of 95% with a margin error of 5% and 27 predictors (main predictors and interaction effects).

### Participants

Community-dwelling older adults, defined as those that live in the community, either alone or with others, and not in the hospital or any institution (e.g., long-term care homes), were participants in this study. Community-dwelling status was self-reported by participants. Community-dwelling older adults were included if they were (a) of age 60 or older; (b) able to ambulate independently with or without an assistive device; (c) able to follow instructions and were cooperative; and (d) could speak, understand, or read English or Igbo. Notably, no participants completed any questionnaire in Igbo. Exclusion criteria were (a) recent cardiovascular events (within the past 6 months), (b) significant hearing or vision loss, (c) had a fracture (within 4 months), and (d) had severe mobility limiting arthritis, vestibular disorder, uncontrolled hypertension, or uncontrolled epilepsy. We obtained verbal and written informed consent to participate from all participants who met the study inclusion criteria.

### Predictive Variables

The predictors used in this study were based on the seven mobility determinants, including physical, financial, cognitive, social, psychological, personal, and environmental factors. See Table 1 for the predictors measured in Waves 1 and 2.

**Table 1.** Predictors Measured in Each Study Wave

Mobility determinant	Wave 1	Wave 2
Cognitive	MoCA	MoCA
Environmental	Modified LEFS	—
Financial	Income	Income
Personal	Age, sex, occupation, education	Age, sex, occupation, education
Physical	Number of comorbidities, BMI, MAP, exercise	Number of comorbidities, BMI, MAP, exercise
Psychological	—	Extraversion, agreeableness, conscientiousness, neuroticism, openness
Social	Living arrangement	Living arrangement

*Notes*: BMI = body mass index; LEFS = Lower Extremity Functional Scale; MAP = mean arterial pressure; MoCA = Montreal Cognitive Assessment Scale.

#### Physical factors

Participants self-reported the number of comorbidities and exercise frequency. Participants’ body mass index (BMI) was calculated using participants’ measured weight and height. Participant’s systolic (SBP) and diastolic (DBP) blood pressure were recorded, and mean arterial pressure (MAP) was calculated using the following formula: MAP = SBP × 1/3 + DBP × 2/3 (Sainas et al., 2016).

#### Cognitive factors

Participants’ cognition was assessed using the Montreal Cognitive Assessment (MoCA), a one-page 30-point test that measures short-term and working memory, executive functioning, attention, and language, and has good internal consistency, with a Cronbach’s alpha on the standardized items of 0.83 (Nasreddine et al., 2005). Higher scores indicate good cognition. The MoCA has been validated in several sub-Saharan African countries, with similar social and cultural contexts to Nigeria (Beath et al., 2018; Masika et al., 2021).

#### Psychological factors

Participants completed the 50-item International Personality Item Pool (IPIP; Hofstee et al., 1992), which was used to assess participants’ personalities based on the five-factor model (extraversion, agreeableness, conscientiousness, neuroticism, and openness to new experience) in Wave 2. The IPIP scale has good internal consistencies (α = 0.88) and is moderately related to the major dimensions of personality (*r* = 0.36–0.67; Ypofanti et al., 2015). The IPIP scale was selected because it has been validated for use in developing countries (Nigeria; Abiola et al., 2012).

#### Environmental factors

We adapted one question (ability to walk a mile) from the Lower Extremity Functional Scale to measure the presence of environmental obstacles (e.g., barriers on the walkway) when walking a mile (Binkley et al., 1999), but only in Wave 1. Participants answered using many barriers, several barriers, some barriers, few barriers, and no barriers.

#### Financial, social, and personal factors

Participants self-reported (a) their level of income (financial factor), (b) the number of people living with them (social factor), and (c) their self-reported age, sex, occupation (skilled, semiskilled, unskilled), and the highest level of education (none, primary, secondary, tertiary; personal factors).

### Outcome Variable: Mobility

#### Performance-based measures

The SPPB assessed older adults’ gait speed, lower extremity physical performance, and balance (Guralnik et al., 1995). The balance test component of the SPPB, consisting of side-by-side stands, semitandem stands, and tandem stand tests, was used to evaluate participants’ balance. The chair stand test component of the SPPB was used to assess the participant’s lower extremity strength, and the time required to complete five sit-to-stand transitions was recorded. The gait speed component of the SPPB, which involves a 4-m walk, was used to evaluate the participant’s gait speed. Participants’ gait speed, balance, and lower extremity strength were rated on a scale of 0–4, with a summary score ranging from 0 to 12, with a higher score indicating better physical performance.

The SPPB is a valid and reliable tool for community-dwelling older adults, as shown by its excellent test–retest reliability (intraclass correlation coefficient values of 0.87–0.89; Freire et al., 2012; Gómez et al., 2013). SPPB scores are moderately correlated with the quadriceps test (*r* > 0.40) and less correlated with the handgrip test (*r* < 0.30), which reinforces convergent and divergent validities (Bernabeu-Mora et al., 2015).

### Self-Reported Measures

A modified self-reported measure of preclinical mobility limitation by Mänty et al. (2007) was used to assess older adults’ self-reported mobility limitations. Participants were asked if they had difficulty (a) walking 2 km, (b) 0.5 km, and (c) climbing up a flight of stairs. Those without mobility limitations with or without task modifications were classified as having no mobility limitation. Older adults facing some difficulty, a great deal of difficulty, or were unable to manage even with assistance were classified as having mobility limitations.

### Data Collection

All assessments were completed in local community halls and meeting areas. Following the obtainment of informed consent, all predictor variables were measured and recorded. Subsequently, participants responded to the self-reported measures of mobility (Manty Preclinical Mobility Limitation Scale) followed by the performance-based measure (SPPB). Participants rested in between the tests as needed. All objective assessments were performed by a trained research assistant while monitored by a licensed physiotherapist.

### Statistical Analyses

Data were analyzed using STATA/IC (v17) with a *p* value for significance set at <.05. The descriptive analysis was presented as means (for continuous variables) and frequency/percentages (for noncontinuous variables). The differences between Waves 1 and 2 in predictors and outcomes were tested using a two-sided *t* test for continuous variables and a chi-square test for noncontinuous variables. We tested the assumptions for regression: normality (Shapiro–Wilk test), heteroskedasticity (Breusch–Pagan), multicollinearity (mean variation inflation factor [VIF]), linearity (scatterplots), and independence (scatterplots). All assumptions were met; for instance, *p* value of >0.05 for normality and heteroskedasticity; for linearity and independence, the scatterplots showed random patterns; for multicollinearity, the mean VIF was <2 for all outcomes (Field, 2013).

We had missing data for income (Wave 1: 35.6%; Wave 2: 39.4%), and Little’s test (Little & Rubin, 1987) was used to test the missing data mechanism indicating that data were missing at random (MCAR; χ^2^[1, 227] = 42.57; *p* = .3201). We performed a single-value regression form of multiple imputations for income and included it in our models (Jakobsen et al., 2017).

Initially, we performed multiple correlation analyses (Spearman’s rank correlation coefficients) to explore the relationships between mobility outcomes and the various multidomain measures. Afterward, three linear regression analyses were performed to determine the associations between predictors and three performance-based mobility outcomes (gait speed, balance, and lower extremity strength). Likewise, three logistic regression analyses were performed to determine the associations between predictors and three self-reported mobility outcomes (Manty Preclinical Mobility Limitation Scale). Two models were generated for each regression in the combined wave. Model 1 contained all predictors. Model 2 started by including all predictors and interactors (only significant interactions). A significant interaction means that the effect of a change in the value of one explanatory variable (e.g., age) on the mean outcome (e.g., balance) depends on the value of another explanatory variable (e.g., MoCA scores; Jaccard et al., 1990). Using a backward elimination approach, the model with the best improved adjusted *R*-square was considered the final model (Model 2).

For Waves 1 and 2, only Model 1 was generated, as there is insufficient power to explore interaction terms. Variables added in Waves 1 and 2 and the combined wave in Model 1 were personal (age, sex, occupation, education, marital status), financial (income), social (living arrangement), physical (number of comorbidities, BMI, MAP, exercise), and cognitive (MoCA score). Additionally, environmental (self-reported presence of obstacle when walking a mile), and psychological variables (extraversion, agreeableness, conscientiousness, neuroticism, and openness) were added to Model 1 for Waves 1 and 2, respectively. We presented only significant predictors of mobility in the combined wave. The tables containing all analyses and for each wave are presented in Supplementary Tables 1–8.

## Results

### Descriptive Statistics

In total, 227 older adults (90 for Wave 1 and 137 for Wave 2) met the criteria for participation in the study. Compared to participants in Wave 1, participants in Wave 2 were younger, worked more skilled occupations, were more educated, were more likely to exercise every day, had lower blood pressure, and had higher cognitive and physical functioning. There were no differences in sex, marital status, BMI, and the number of people living with older adults in Waves 1 and 2. Table 2 describes the two waves’ demographics, predictors, and outcomes, and the differences between Waves 1 and 2.

**Table 2.** Demographic Characteristics of the Older Adults (*n* = 227)

Participant characteristics	Wave 1 (*n* = 90)		Wave 2 (*n* = 137)		Statistical test^d^
	Mean (*SD*)	*n* (%)	Mean (*SD*)	*n* (%)	
Age (years)	68.9 (7.9)		65.1 (5.4)		*t*(225) = 4.23, *p* < .001
Sex (female)		55 (61.1)		69 (50.4)	χ^2^(1) = 2.53, *p* = .112
Occupation^a^					χ^2^(2) = 28.31, *p* < .001
Skilled		8 (8.9)		51 (37.2)	
Semiskilled		5 (5.6)		15 (10.9)	
Unskilled		74 (82.2)		67 (48.9)	
Missing data		3 (3.3)		4 (2.9)	
Education					χ^2^(3) = 30.85, *p* < .001
No educational qualification		25 (27.8)		26 (19.0)	
Primary		49 (54.4)		38 (27.7)	
Secondary		9 (10.0)		42 (30.7)	
Tertiary		6 (6.7)		31 (22.6)	
Missing data		1 (1.1)		—	
Marital status					χ^2^(1) = 0.01, *p* = .914
Married		62 (68.9)		96 (70.1)	
Widowed/divorced/single		25 (27.8)		40 (29.2)	
Missing data		3 (3.3)		1 (0.7)	
Monthly household income^b^					*t*(139) = −5.88, *p* < .001
Below poverty line		44 (48.9)		31 (22.6)	
Above poverty line		14 (15.6)		52 (38.0)	
Missing data		32 (35.6)		54 (39.4)	
Comorbidities					*t*(224) = −2.29, *p* = .023
0–2 conditions		75 (83.3)		123 (89.8)	
>2 conditions		14 (15.6)		14 (10.2)	
Missing data		1 (1.1)		—	
Frequency of exercise					χ^2^(1) = 116.93, *p* < .001
None		63 (70.0)		8 (5.8)	
Daily		9 (10.0)		83 (60.6)	
Weekly		5 (5.6)		17 (12.4)	
Other		6 (6.7)		28 (20.4)	
Missing data		7 (7.8)		1 (0.7)	
Body mass index	25.2 (6.7)		26.4 (5.4)		*t*(223) = −1.39, *p* = .166
Mean arterial pressure	104.7 (19.9)		96.1 (14.7)		*t*(224) = 3.71, *p* < .001
MoCA score	16.2 (7.1)		22.6 (6.3)		*t*(222) = −7.00, *p* < .001
Living arrangement	4.8 (3.6)		4.9 (2.6)		*t*(223) = −0.22, *p* = .823
SPPB total score^c^	8.63 (3.32)		9.66 (1.98)		*t*(225) = −2.93, *p* = .004
4-m gait speed	3.49 (0.90)		3.25 (0.95)		
Balance	3.67 (0.84)		3.75 (0.67)		
Five times sit-to-stand	2.10 (1.34)		2.68 (1.08)		
Manty 2-km walk					χ^2^(1) = 20.93, *p* < .001
No limitation		41 (45.6)		70 (51.1)	
Limitation		46 (51.1)		17 (12.4)	
Missing data		3 (3.3)		43 (31.4)	
Manty 0.5-km walk					χ^2^ (1) = 15.17, *p* < .001
No limitation		47 (52.2)		71 (51.8)	
Limitation		40 (44.4)		16 (11.7)	
Missing data		3 (3.3)		43 (31.4)	
Manty stair climb					χ^2^(1) = 28.40, *p* < .001
No limitation		30 (33.3)		65 (47.4)	
Limitation		57 (63.3)		22 (16.1)	
Missing data		3 (3.3)		43 (31.4)	

*Notes*: MoCA = Montreal Cognitive Assessment scale; *SD* = standard deviation; SPPB = Short Physical Performance Battery; *t* = *t*-statistic; χ^2^ = chi-square statistic.

Poverty line was set at the adjusted $1.90/day.

Occupational classification: skilled = civil servants (retired and active), mechanical engineer, teacher, barrister; semiskilled = vulcanizer, tailor, carpenter, hotel supervisor, builder, welder, self-employed, business owner; unskilled = cleaner, farmer, trader, security officer, gateman, none.

SPPB scores ranged from 0 to 12, with higher scores indicating higher physical performance. Each subset, including gait speed, balance, and chair rise test, scored 0 to 4, with 4 indicating the highest performance in each subset.

Statistical tests comparing Waves 1 and 2.

### Correlation Analyses

Table 3 summarizes the results of the correlation analyses for the combined data set. Age, the number of comorbidities, MoCA score, and the highest level of education showed the largest number and strongest correlations with performance-based and self-reported mobility measures, highlighting the importance of multiple domains in determining mobility. However, the strength of these correlations was poor to fair. For bivariate correlations for Wave 1 and Wave 2, see Supplementary Tables 1 and 2.

**Table 3.** Bivariate Correlations of Study Variables in the Combined Wave

Variable	1	2	3	4	5	6	7	8	9	10	11	12	13	14	15	16	17	18
1. Age	—																	
2. Sex	−0.05	—																
3. Income	−0.29^*^	−0.35^*^	-															
4. Living arrangement	0.00	−0.09	0.11	—														
5. Comorbidities ^a^	0.08	0.19^*^	−0.39^*^	0.00	—													
6. MoCA	−0.31^*^	−0.34^*^	0.39^*^	0.15^*^	−0.18^*^	—												
7. BMI	−0.03	0.13	0.17^*^	0.06	−0.07	0.16^*^	—											
8. MAP	0.08	0.05	−0.19^*^	−0.10	0.16^*^	−0.19^*^	0.26^*^	—										
9. Occupation	0.28^*^	0.38^*^	−0.43^*^	−0.06	0.16^*^	−0.40^*^	−0.03	0.14^*^	—									
10. Education	−0.32^*^	−0.32^*^	0.59^*^	0.17^*^	−0.25^*^	0.57^*^	0.20^*^	−0.12	−0.48^*^	—								
11. Marital status	0.09	0.44^*^	−0.27^*^	−0.10	0.34^*^	−0.24^*^	−0.02	0.06	0.32^*^	—0.38^*^	—							
12. Exercise^b^	−0.02	0.02	0.01	0.02	−0.04	−0.03	0.01	−0.03	0.06	−0.04	0.00	—						
13. Balance^c^	−0.33^*^	−0.04	0.07	−0.05	−0.21^*^	**0.18** ^*^	0.05	−0.13	−0.12	**0.14** ^*^	−0.09	−0.02	—					
14. Gait speed^c^	−0.32^*^	−0.12	**0.27** ^*^	0.00	−0.26^*^	0.13	0.13	−0.11	−0.17^*^	**0.37** ^*^	−0.27^*^	−0.02	**0.38** ^*^	—				
15. Chair stands^c^	−0.38^*^	−0.14^*^	**0.23** ^*^	−0.03	−0.15^*^	**0.28** ^*^	−0.04	−0.14^*^	−0.28^*^	**0.32** ^*^	−0.19^*^	−0.02	**0.34** ^*^	**0.30** ^*^	—			
16. Manty walk 2 km	**0.19** ^*^	**0.21** ^*^	−0.17	0.10	**0.16** ^*^	−0.26^*^	0.08	0.06	**0.20** ^*^	−0.22^*^	0.06	0.04	−0.19^*^	−0.11	−0.29^*^	—		
17. Manty walk 0.5 km	**0.22** ^*^	0.14	−0.12	0.11	**0.21** ^*^	−0.21^*^	0.03	0.01	**0.20** ^*^	−0.18^*^	0.09	0.06	−0.18^*^	−0.11	−0.30^*^	**0.86** ^*^	—	
18. Manty stair climb	0.12	**0.34** ^*^	−0.25^*^	0.05	**0.24** ^*^	−0.27^*^	0.01	0.01	0.12	−0.15	0.06	−0.02	−0.20^*^	−0.05	−0.26^*^	**0.59** ^*^	**0.58** ^*^	—

*Notes*: BMI = body mass index; MAP = mean arterial pressure; MoCA = Montreal Cognitive Assessment.

Number of comorbidities.

Frequency of exercise (self-report).

Scores from balance, gait speed, and lower limb strength components of the Short Physical Performance Battery.

*p* value <0.05 (two-tailed). All * are significant.

### Predictors of Performance-Based Mobility Outcomes

Physical factors (BMI, number of comorbidities, MAP, and no exercise) and personal factors (age and education) were significant predictors of gait speed (Model 1). In Model 2, cognitive factors (MOCA scores) became significant predictors; education was no longer a significant predictor, but the interactions between age and living arrangement and MoCA and living arrangement were significant predictors of gait speed (adjusted *R*^2^ from 26.7% to 31.3%). See Table 4.

**Table 4.** Significant Predictors of Community-Dwelling Older Adults’ Gait Speed (SPPB Walk Test)

Variable	Model 1			Model 2		
	*B* (95% CI)	β	*p* Value	*B* (95% CI)	β	*p* Value
Age	−0.043 (−0.063; −0.022)	−0.300	<.001	−0.027 (−0.049; −0.005)	−0.192	.016
Number of comorbidities	−0.149 (−0.270; −0.029)	−0.174	.015	−0.134 (−0.246; −0.022)	−0.157	.020
BMI	0.027 (0.004; 0.049)	0.161	.021	.029 (.007;.050)	0.176	.009
MAP	−0.008 (−0.015; 0.000)	−0.146	.037	−0.009 (−0.016; −0.002)	−0.163	.017
Education (primary)	0.484 (0.123; 0.844)	0.249	.009	—	—	—
Education (secondary)	0.718 (0.253; 1.183)	0.329	.003	—	—	—
Education (tertiary)	0.644 (0.074; 1.213)	0.252	.027	—	—	—
Exercise (not at all)	0.380 (0.073; 0.688)	0.184	.016	0.379 (0.081; 0.678)	0.183	.013
MoCA	—	—	—	−0.050 (−0.082; −0.019)	−0.370	.002
Age × Living arrangement	—	—	—	−0.002 (−0.004; −0.001)	−0.523	.001
MoCA × Living arrangement	—	—	—	0.007 (0.002;0.012)	0.552	.004
Adjusted *R*^2^	0.267			0.313		

*Notes*: β = standardized beta; *B* = unstandardized beta; BMI = body mass index; CI = confidence interval; MAP = mean arterial pressure; MoCA = Montreal Cognitive Assessment; SPPB = Short Physical Performance Battery. Model 1 contains all predictors: age, sex, income, occupation, education, martial status, exercise, living arrangement, number of comorbidities, MoCA score, BMI, and MAP. All predictors in Model 1 were initially entered into Model 2 along with significant interactors. Predictors were selected using backwards stepwise regression to maximize adjusted *R*^2^. Model 2 includes age, sex, occupation, education, martial status, exercise, number of comorbidities, MoCA score, BMI, MAP, age × living arrangement, martial status × education, MoCA × living arrangement. Significance threshold was set at *p* value of <.05.

Although only age and number of comorbidities significantly predicted balance in Model 1, several significant interaction terms (age × MoCA, number of comorbidities × tertiary education, widowed/divorced/single × no exercise, widowed/divorced/single × other exercise frequencies, MAP × other exercise frequencies) had predictive ability in Model 2 (adjusted *R*^2^ from 11.2% to 27.7%). See Table 5.

**Table 5.** Significant Predictors of Community-Dwelling Older Adults’ Balance (SPPB Balance Test)

Variable	Model 1			Model 2		
	*B* (95% CI)	β	*p* Value	*B* (95% CI)	β	*p* Value
Age	−0.042 (−0.059; −0.024)	−0.381	<.001	−0.056 (−0.096; −0.016)	−0.515	.006
Number of comorbidities	−0.149 (−0.252; −0.046)	−0.230	.005	−0.325 (−0.525; −0−0.125)	−0.504	.002
MoCA	—	—	—	−0.148 (−0.276; −0.020)	−1.410	.023
MAP	—	—	—	−0.010 (−0.019; −0.001)	−0.246	.024
Marital status (widowed/divorced/single)	—	—	—	−0.367 (−0.693; −0.041)	−0.227	.027
Exercise (others)	—	—	—	−3.221 (−5.238; −1.205)	−1.584	.002
Age × MoCA	—	—	—	0.002 (0.000; 0.004)	1.291	.030
Number of comorbidities × Education (tertiary)	—	—	—	0.333 (0.032; 0.634)	0.214	.030
Marital status (widowed/divorced/single) × exercise (not at all)	—	—	—	0.771 (0.298; 1.243)	0.298	.002
Marital status (widowed/ divorced/single) × Exercise (others)	—	—	—	0.972 (0.381; 1.562)	0.273	.001
MAP × Exercise (others)	—	—	—	0.028 (0.008; 0.049)	1.292	.007
Adjusted *R*^2^	0.112			0.277		

*Notes*: β = standardized beta; B = unstandardized beta; BMI = body mass index; MAP = mean arterial pressure; MoCA = Montreal Cognitive Assessment; SPPB = Short Physical Performance Battery. Model 1 contains all predictors: age, sex, income, occupation, education, martial status, exercise, living arrangement, number of comorbidities, MoCA score, BMI, and MAP. All predictors in Model 1 were initially entered into Model 2 along with significant interactors. Predictors were selected using backwards stepwise regression to maximize adjusted *R*^2^. Model 2 includes: age, income, education, martial status, exercise, living arrangement, number of comorbidities, MoCA score, MAP, age × living arrangement, age × MoCA, number of comorbidities × education, exercise × marital status, exercise × living arrangement, living arrangement × MoCA, MAP × exercise. Significance threshold was set at *p* value of <.05.

In Model 1, age was associated with older adults’ lower extremity strength (Model 1). In Model 2, the interactions between living arrangement and weekly exercise, and living arrangement and MoCA score improved the model (adjusted *R*^2^ from 15.7% to 23.0%). See Table 6.

**Table 6.** Significant Predictors of Community-Dwelling Older Adults’ Lower Extremity Strength (SPPB Chair Stand Test)

Variable	Model 1			Model 2		
	*B* (95% CI)	β	*p* value	*B* (95% CI)	β	*p* Value
Age	−0.050 (−0.078; −0.021)	−0.277	.001	−0.041 (−0.066; −0.015)	−0.225	.002
Living arrangement × Exercise (Weekly)	—	—	—	−0.241 (−0.439; −0.044)	−0.329	.017
Living arrangement × MoCA	—	—	—	0.012 (0.003; 0.020)	.703	.006
Adjusted *R*^2^	0.157			0.230		

*Notes*: β = standardized beta; *B* = unstandardized beta; MoCA = Montreal Cognitive Assessment. Model 1 contains all predictors: age, sex, income, occupation, education, martial status, exercise, living arrangement, number of comorbidities, MoCA score, BMI, and MAP. All predictors in Model 1 were initially entered into Model 2 along with significant interactors. Predictors were selected using backwards stepwise regression to maximize adjusted *R*^2^. Model 2 includes: age, occupation, marital status, exercise, living arrangement, MoCA score, living arrangement × exercise, living arrangement × MoCA, living arrangement × MAP. Significance threshold was set at *p* value of <.05.

### Predictors of Self-Reported Mobility Outcomes

Sex (female) and no exercise significantly predicted self-reported inability to walk 2 km (Model 1). In Model 2, sex was no longer significant; however, no exercise remains significant, and the number of comorbidities and the interaction between sex and secondary education became significant predictors (pseudo *R*^2^ from 18.4% to 25.8%).

The number of comorbidities and no exercise predicted self-reported inability to walk 0.5 km (Model 1). In Model 2, living arrangement, the interaction between BMI and living arrangement, and the interaction between sex and education were significant predictors of mobility limitation in walking 0.5 km (pseudo *R*^2^ from 16.1% to 30.9%).

Female sex, number of comorbidities, and no exercise were significant predictors of self-reported inability to climb stairs (Model 1). In Model 2, sex and lack of exercise were no longer significant; however, the interaction between education and living arrangement, and education and exercise became significant (pseudo *R*^2^ from 27.9% to 39.5%). See Table 7.

**Table 7.** Significant Predictors of Community-Dwelling Older Adults’ Self-Reported Mobility Limitations (Manty Preclinical Mobility Limitation Scale)

Variable	Model 1		Model 2	
	*B* (95% CI)	*p* Value	*B* (95% CI)	*p* Value
Inability to walk 2 km				
** **Female sex	1.262 (0.054; 2.470)	.041	—	—
** **Exercise (not at all)	1.094 (0.129; 2.059)	.026	1.295 (0.260; 2.331)	.014
** **Number of comorbidities	—	—	0.387 (0.026; 0.748)	.036
** **Female sex × Education (secondary)	—	—	−3.658 (−7.059; −0.257)	.035
** **Pseudo *R*^2^	0.184		0.258	
Inability to walk 0.5 km				
** **Number of comorbidities	0.380 (0.002; 0.758)	.049	0.452 (0.082;0.823)	.017
** **Exercise (not at all)	1.010 (0.015; 2.005)	.047	1.401 (0.332; 2.470)	.010
** **Living arrangement	—	—	−2.945 (−5.003; −0.887)	.005
** **BMI × Living arrangement	—	—	0.037 (0.004; 0.070)	.026
** **Female sex × Education (secondary)	—	—	−4.966 (−8.666; −1.266)	.009
** **Pseudo *R*^2^	0.161		0.309	
Inability to climb one flight of stairs				
** **Female sex	2.325 (1.044; 3.606)	<.001	—	—
** **Number of comorbidities	0.421 (0.015; 0.827)	.042	0.516 (0.021; 1.012)	.041
** **Exercise (not at all)	1.881 (0.851; 2.911)	<.001	—	—
** **Education (secondary) × Living arrangement	—	—	−1.742 (−3.205; −0.279)	.020
** **Education (primary) × Exercise (not at all)	—	—	2.562 (0.107; 5.016)	.041
** **Education (secondary) × Exercise (others)	—	—	7.456 (0.009; 14.903)	.049
** **Pseudo *R*^2^	0.279		0.395	

*Notes*: β = standardized beta; *B* = unstandardized beta; BMI = body mass index; CI = confidence interval; MAP = mean arterial pressure; MoCA = Montreal Cognitive Assessment. Model 1 contains all predictors: age, sex, income, occupation, education, martial status, exercise, living arrangement, number of comorbidities, MoCA score, BMI, and MAP. All predictors in Model 1 were initially entered into Model 2 along with significant interactors. Predictors were selected using backwards stepwise regression to maximize pseudo *R*^2^. Model 2 predicting inability to walk 2 km includes: age, sex, education, martial status, exercise, living arrangement, number of comorbidities, MoCA score, BMI, and sex × education. Model 2 predicting inability to walk 0.5 km includes: age, sex, education, exercise, living arrangement, number of comorbidities, MoCA score, BMI, MAP, age × living arrangement, sex × education, and living arrangement × BMI. Model 2 predicting inability to climb 1 flight of stairs includes: age, sex, income, occupation, education, marital status, exercise, living arrangement, number of comorbidities, MoCA score, BMI, MAP, sex × education, education × living arrangement, and education × exercise. Significance threshold was set at *p* value of <.05.

## Discussion

Our study evaluated the ability of cognitive, environmental, financial, personal, physical, psychological, and social factors to predict mobility among community-dwelling older adults in Nigeria. To the best of our knowledge, this is the first study that tests Webber et al.’s (2010) conical model of mobility among community-dwelling older adults in a developing nation and simultaneously assesses the predictors of performance-based (gait speed, balance, and lower extremity function) and self-reported (inability to walk 0.5 km, 2 km, and stair climb) mobility outcomes. In Model 2 (i.e., model with interaction effect), none of the seven determinants of mobility were predictors of mobility in all outcomes measured. However, the number of comorbidities (physical factor) significantly predicted mobility in five (gait speed, balance, inability to walk 0.5 km, 2 km, and stair climb) out of the six outcomes in the final model. Age (personal factor) was significantly associated with all performance-based mobility outcomes in the expected direction—an increase in age was associated with a lower score in performance-based mobility outcomes. In addition, individuals with a history of no exercise (physical factor) have a higher odd of self-reporting an inability to walk 0.5 or 2 km. We discuss the study findings with the existing literature that has tested or expanded Webber’s model.

The findings of our study partially differ from previous studies exploring Webber et al.’s (2010) model in the context of performance-based mobility outcomes. Similar to our study, in their pooled data set, Giannouli et al. (2019) found that physical factors (muscle strength) and age were significant predictors of performance-based mobility among community-dwelling older adults. However, no cognitive factors were significant in their pooled data set, whereas our pooled analyses found that older adults’ MoCA scores were associated with mobility. This difference between our study and Giannouli et al. could be due to how cognitive factors were assessed between studies. Giannouli et al. captured cognitive factors by assessing participants’ planning ability, visuospatial attention, and spatial working memory. The MoCA assesses short-term and working memory, executive functioning, attention, and language to comprehensively assess the older adult’s global cognition. Another possible reason could be how the performance-based mobility outcomes were assessed in both studies. Although we observed our participants performing the mobility test in a laboratory setting, Giannouli et al. captured “real-life” performance-based mobility using smartphone motion and GPS data. Evidence has shown that laboratory-based mobility tests, such as gait speed from a 4- or 3-m walk test, do not equate to the real-life gait speed capacity captured via smartphone technologies (Giannouli et al., 2016). Therefore, when possible, using smartphone technologies of GSP to capture the gait speed of community older adults is encouraged.

Conversely, our study’s findings for self-reported mobility measures are predominantly congruent with other studies exploring Webber et al.’s model. Factors that were predictors of self-reported mobility across Meyer et al.’s (2014) and Jafari et al.’s (2020) studies and our study were physical (number of comorbidities, physical activity/exercise), personal (sex/gender), and social (social network/social activity). Although our study and Jafari et al. (2020) reported that education was a predictor, this factor was not retained in the final model in Meyer et al.’s (2014) study. Other factors, such as cognition, age, and marital status, were significant predictors of community and personal mobility (Meyer et al., 2014) and mobility limitation (Jafari et al., 2020); these factors were not in our study. Several reasons would have explained the differences in the findings across these three studies, including but not limited to how each determinant and self-reported mobility outcome were measured in each study. Our study captured self-reported mobility on the inability to walk 0.5 or 2 km or inability to climb a flight of stairs, whereas Meyer et al. (2014) and Jafari et al. (2020) created composite scores of 10 questions relating to walking several distances and other mobility-related activities involving upper limbs such as the ability to reach arms, pull and push objects (carrying groceries). More so, our study included the interaction effect in our model, highlighting that some determinants’ predictive factors depend on another determinant. For instance, females are more likely to report the inability to walk 2 km than males; however, this is not true when educational levels are considered in such a model—this highlights the importance of interactions between mobility determinants to explain the complexity of factors influencing mobility.

Living arrangement, education, and exercise are the interacting variables (defined as predicting variables that interact with each other to produce an interaction effect) in our study. Across these interacting variables, living arrangements consistently and significantly interact with other variables to predict mobility outcomes. As shown in our study, the predictive power of some of the determinants, for instance, age, cognition status, and the number of exercises on mobility outcome, such as gait speed, may depend on an individual’s living arrangement. This finding highlights the critical role of social factors in predicting mobility outcomes, as noted in a previous study. Kuspinar et al. (2020) reported that social support (a social factor) alongside driving and gait speed has the strongest effect on life-space mobility among community-dwelling older Canadians. Older adults have reported the importance of social interactions that can positively influence their community mobility (Gardner, 2014). Similarly, evidence has shown that older adults with more extensive social networks have a higher level of mobility (McLaughlin et al., 2011). This finding has practice implications. Current interventions aimed to promote mobility among older adults in most developing countries, including Nigeria, predominantly focus on enhancing the physical determinants, such as muscle strength. Social factors, such as living together, are inherently embedded in Nigerian culture and hence are not objectively considered a critical area to improve mobility. We argued that amidst competing resources in developing countries, interventions to improve social factors, such as living arrangements or social networks, are recommended, as such intervention effect may influence other mobility determinants, ultimately enhancing mobility. Future studies would explore if interventions that focused on improving social outcomes, such as social networks or living arrangements, would provide a ripple effect on other mobility determinants improving older adults’ mobility.

Our study boasts several strengths and limitations. Our study is the first to evaluate Webber et al.’s conical model of mobility among community-dwelling older adults in a developing nation (Nigeria) using both performance-based and self-reported mobility measures simultaneously. The addition of interactor terms improves the overall predictive ability of the regression model and allows for a greater understanding of the relationships between mobility determinants and their impacts. However, because of the limited sample size, we consider this finding exploratively preliminary; therefore, interpretation should be cautioned. Another limitation of our study is that participants in each wave were recruited using a stratified sampling approach, which may cause an under-or-over presentation of the population, causing biases in the results and compromising our ability to generalize our findings. Other limitations include the cross-sectional design of our study, which does not allow for causal conclusions and the use of self-reported measures for several predictors (e.g., number of comorbidities, living arrangement, exercise). Also, environmental and psychological factors were missing in the combined wave but were included in Waves 1 and 2, respectively. Additionally, we measured the environmental factors by modifying a question from the LEFS scale, rather than traditional environmental measures (e.g., street connectivity or land mass use). The lack of reliability and validity of the modified question may have influenced our findings. Finally, the threshold for significance in our regression models was not corrected for multiple testing, and thus, there may be false positives present in our regression analyses, even though we accounted for the interaction effects in our sample size calculation. We recommend further studies to explore cross-sectionally and longitudinally the interaction effects rather than the individual additive effects of mobility determinants using a larger sample size.

## Conclusion

Community-dwelling older adults with greater numbers of comorbidities had more restricted performance-based and self-reported mobility. Older age was associated with poorer performance-based mobility, whereas a history of no exercise was associated with poorer self-reported mobility. The number of comorbidities was associated with poorer mobility across both self-reported and performance-based measurements. The factors affecting older adults’ mobility are complex; our findings build upon Webber et al.’s conical model of mobility and add to the existing literature surrounding the determinants of mobility among older adults residing in a developing nation, such as Nigeria. A longitudinal study utilizing a large data set is recommended to establish a cause-and-effect relationship between mobility determinants and older adults’ mobility in developing regions.

## Supplementary Material

igad019_suppl_Supplementary_Material

## References

[CIT0001] Abiola, T., Udofia, O., & Yunusa, S. (2012). Reliability and concurrent validity of the International Personality Item Pool (IPIP) Big-Five factor markers in Nigeria. Nigerian Journal of Psychiatry, 10(2), 38. https://www.ajol.info/index.php/njpsyc/article/view/113002

[CIT0002] African Academy of Sciences. (2020). *Addressing long term care for Nigeria’s aging population*. https://www.aasciences.africa/news/addressing-long-term-care-nigerias-aging-population

[CIT0003] Bean, J. F., Olveczky, D. D., Kiely, D. K., LaRose, S. I., & Jette, A. M. (2011). Performance-based versus patient-reported physical function: What are the underlying predictors? Physical Therapy, 91(12), 1804–1811. doi:10.2522/ptj.20100417 22003163 PMC3229045

[CIT0004] Beard, J. R., Officer, A., de Carvalho, I. A., Sadana, R., Pot, A. M., Michel, J. P., Lloyd-Sherlock, P., Epping-Jordan, J. E., Peeters, G., Mahanani, W. R., Thiyagarajan, J. A., & Chatterji, S. (2016). The world report on ageing and health: A policy framework for healthy ageing. Lancet, 387(10033), 2145–2154. doi:10.1016/S0140-6736(15)00516-4 26520231 PMC4848186

[CIT0005] Beath, N., Asmal, L., van den Heuvel, L., & Seedat, S. (2018). Validation of the Montreal cognitive assessment against the RBANS in a healthy South African cohort. The South African Journal of Psychiatry, 24, 1304. doi:10.4102/sajpsychiatry.v24i0.1304 30349744 PMC6191746

[CIT0006] Bernabeu-Mora, R., Medina-Mirapeix, F., Llamazares-Herrán, E., García-Guillamón, G., Giménez-Giménez, L. M., & Sánchez-Nieto, J. M. (2015). The Short Physical Performance Battery is a discriminative tool for identifying patients with COPD at risk of disability. International Journal of Chronic Obstructive Pulmonary Disease, 10, 2619–2626. doi:10.2147/COPD.S94377 26664110 PMC4671756

[CIT0007] Binkley, J. M., Stratford, P. W., Lott, S. A., & Riddle, D. L. (1999). The Lower Extremity Functional Scale (LEFS): Scale development, measurement properties, and clinical application. North American Orthopaedic Rehabilitation Research Network. Physical Therapy, 79(4), 371–383. doi:10.1093/ptj/79.4.371 10201543

[CIT0008] Dunlap, P. M., Rosso, A. L., Zhu, X., Klatt, B. N., & Brach, J. S. (2021). The association of mobility determinants and life space among older adults. The Journals of Gerontology, Series A: Biological Sciences and Medical Sciences, 77(11), 2320–2328. doi:10.1093/gerona/glab268 PMC9678196 34529773

[CIT0009] Field, A. (2013). Discovering statistics using IBM SPSS statistics (4th ed.). Sage Publications Ltd.

[CIT0010] Freire, A. N., Guerra, R. O., Alvarado, B., Guralnik, J. M., & Zunzunegui, M. V. (2012). Validity and reliability of the short physical performance battery in two diverse older adult populations in Quebec and Brazil. Journal of Aging and Health, 24(5), 863–878. doi:10.1177/0898264312438551 22422762

[CIT0011] Gardner, P. (2014). The role of social engagement and identity in community mobility among older adults aging in place. Disability and Rehabilitation, 36(15), 1249–1257. doi:10.3109/09638288.2013.837970 24099580

[CIT0012] Gayman, M. D., Turner, R. J., & Cui, M. (2008). Physical limitations and depressive symptoms: Exploring the nature of the association. The Journals of Gerontology, Series B: Psychological Sciences and Social Sciences, 63(4), S219–S228. doi:10.1093/geronb/63.4.s219 18689771 PMC2844725

[CIT0013] Giannouli, E., Bock, O., Mellone, S., & Zijlstra, W. (2016). Mobility in old age: Capacity is not performance. Biomed Research International, 2016, 3261567. doi:10.1155/2016/3261567 27034932 PMC4789440

[CIT0014] Giannouli, E., Fillekes, M. P., Mellone, S., Weibel, R., Bock, O., & Zijlstra, W. (2019). Predictors of real-life mobility in community-dwelling older adults: An exploration based on a comprehensive framework for analyzing mobility. European Review of Aging and Physical Activity, 16, 19. doi:10.1186/s11556-019-0225-2 31700551 PMC6825723

[CIT0015] Gómez, J. F., Curcio, C. L., Alvarado, B., Zunzunegui, M. V., & Guralnik, J. (2013). Validity and reliability of the Short Physical Performance Battery (SPPB): A pilot study on mobility in the Colombian Andes. Colombia Medica, 44(3), 165–171. doi:10.25100/cm.v44i3.1181 24892614 PMC4002038

[CIT0016] Guralnik, J. M., Ferrucci, L., Simonsick, E. M., Salive, M. E., & Wallace, R. B. (1995). Lower-extremity function in persons over the age of 70 years as a predictor of subsequent disability. The New England Journal of Medicine, 332(9), 556–561. doi:10.1056/NEJM199503023320902 7838189 PMC9828188

[CIT0017] Hofstee, W. K., de Raad, B., & Goldberg, L. R. (1992). Integration of the Big Five and circumplex approaches to trait structure. Journal of Personality and Social Psychology, 63(1), 146–163. doi:10.1037//0022-3514.63.1.146 1494982

[CIT0018] Jaccard, J., Wan, C. K., & Turrisi, R. (1990). The detection and interpretation of interaction effects between continuous variables in multiple regression. Multivariate Behavioral Research, 25(4), 467–478. doi:10.1207/s15327906mbr2504_4 26820822

[CIT0019] Jakobsen, J. C., Gluud, C., Wetterslev, J., & Winkel, P. (2017). When and how should multiple imputation be used for handling missing data in randomised clinical trials—A practical guide with flowcharts. BMC Medical Research Methodology, 17(1), 162. doi:10.1186/s12874-017-0442-1 29207961 PMC5717805

[CIT0020] Jansen, C. P., Diegelmann, M., Schnabel, E. L., Wahl, H. W., & Hauer, K. (2017). Life-space and movement behavior in nursing home residents: Results of a new sensor-based assessment and associated factors. BMC Geriatrics, 17(1), 36. doi:10.1186/s12877-017-0430-7 28129741 PMC5273820

[CIT0021] Jafari, A., Aminisani, N., Shamshirgaran, S. M., Rastgoo, L., & Gilani, N. (2020). Predictors of mobility limitation in older adults: A structural equation modeling analysis. Baltic Journal of Health and Physical Activity, 12(1), 3.

[CIT0022] Kuspinar, A., Verschoor, C. P., Beauchamp, M. K., Dushoff, J., Ma, J., Amster, E., Bassim, C., Dal Bello-Haas, V., Gregory, M. A., Harris, J. E., Letts, L., Neil-Sztramko, S. E., Richardson, J., Valaitis, R., & Vrkljan, B. (2020). Modifiable factors related to life-space mobility in community-dwelling older adults: Results from the Canadian Longitudinal Study on Aging. BMC Geriatrics, 20(1), 35. doi:10.1186/s12877-020-1431-5 32005107 PMC6995110

[CIT0023] Little, R. J. A., & Rubin, D. B. (1987). Statistical analysis with missing data. John Wiley & Sons.

[CIT0024] Mänty, M., Heinonen, A., Leinonen, R., Törmäkangas, T., Sakari-Rantala, R., Hirvensalo, M., von Bonsdorff, M. B., & Rantanen, T. (2007). Construct and predictive validity of a self-reported measure of preclinical mobility limitation. Archives of Physical Medicine and Rehabilitation, 88(9), 1108–1113. doi:10.1016/j.apmr.2007.06.016 17826454

[CIT0025] Masika, G. M., Yu, D. S. F., & Li, P. W. C. (2021). Accuracy of the Montreal Cognitive Assessment in detecting mild cognitive impairment and dementia in the rural African population. Archives of Clinical Neuropsychology, 36(3), 371–380. doi:10.1093/arclin/acz086 31942599

[CIT0026] McLaughlin, D., Adams, J. O. N., Vagenas, D., & Dobson, A. (2011). Factors which enhance or inhibit social support: A mixed-methods analysis of social networks in older women. Ageing & Society, 31(1), 18–33. https://psycnet.apa.org/doi/10.1017/S0144686X10000668

[CIT0027] Nasreddine, Z. S., Phillips, N. A., Bédirian, V., Charbonneau, S., Whitehead, V., Collin, I., Cummings, J. L., & Chertkow, H. (2005). The Montreal Cognitive Assessment, MoCA: A brief screening tool for mild cognitive impairment. Journal of the American Geriatrics Society, 53(4), 695–699. doi:10.1111/j.1532-5415.2005.53221.x 15817019

[CIT0028] Nielsen, L. M., Kirkegaard, H., Østergaard, L. G., Bovbjerg, K., Breinholt, K., & Maribo, T. (2016). Comparison of self-reported and performance-based measures of functional ability in elderly patients in an emergency department: Implications for selection of clinical outcome measures. BMC Geriatrics, 16(1), 199. doi:10.1186/s12877-016-0376-1 27899065 PMC5129645

[CIT0029] Olawole, M. O., & Aloba, O. (2014). Mobility characteristics of the elderly and their associated level of satisfaction with transport services in Osogbo, Southwestern Nigeria. Transport Policy, 34, 105–116. doi:10.1016/j.tranpol.2014.05.018

[CIT0030] Perkins, J. M., Multhaup, K. S., Perkins, H. W., & Barton, C. (2008). Self-efficacy and participation in physical and social activity among older adults in Spain and the United States. Gerontologist, 48(1), 51–58. doi:10.1093/geront/48.1.51 18381832

[CIT0031] Roedersheimer, K. M., Pereira, G. F., Jones, C. W., Braz, V. A., Mangipudi, S. A., & Platts-Mills, T. F. (2016). Self-reported versus performance-based assessments of a simple mobility task among older adults in the emergency department. Annals of Emergency Medicine, 67(2), 151–156. doi:10.1016/j.annemergmed.2015.07.007 26238786 PMC4724447

[CIT0032] Sager, M. A., Dunham, N. C., Schwantes, A., Mecum, L., Halverson, K., & Harlowe, D. (1992). Measurement of activities of daily living in hospitalized elderly: A comparison of self-report and performance-based methods. Journal of the American Geriatrics Society, 40(5), 457–462. doi:10.1111/j.1532-5415.1992.tb02011.x 1634697

[CIT0033] Sainas, G., Milia, R., Palazzolo, G., Ibba, G., Marongiu, E., Roberto, S., Pinna, V., Ghiani, G., Tocco, F., & Crisafulli, A. (2016). Mean blood pressure assessment during post-exercise: Result from two different methods of calculation. Journal of Sports Science & Medicine, 15(3), 424–433. https://www.jssm.org/jssm-15-424.xml 27803621 PMC4974855

[CIT0034] Saka, S., Oosthuizen, F., & Nlooto, M. (2019). National policies and older people’s healthcare in sub-Saharan Africa: A scoping review. Annals of Global Health, 85(1), 91. doi:10.5334/aogh.2401 31251482 PMC6634477

[CIT0035] Suzman, R., Beard, J. R., Boerma, T., & Chatterji, S. (2015). Health in an ageing world—What do we know? Lancet, 385(9967), 484–486. doi:10.1016/S0140-6736(14)61597-X 25468156

[CIT0036] Ullrich, P., Eckert, T., Bongartz, M., Werner, C., Kiss, R., Bauer, J. M., & Hauer, K. (2019). Life-space mobility in older persons with cognitive impairment after discharge from geriatric rehabilitation. Archives of Gerontology and Geriatrics, 81, 192–200. doi:10.1016/j.archger.2018.12.007 30605862

[CIT0037] Umstattd Meyer, M. R., Janke, M. C., & Beaujean, A. A. (2014). Predictors of older adults’ personal and community mobility: Using a comprehensive theoretical mobility framework. Gerontologist, 54(3), 398–408. doi:10.1093/geront/gnt054 23749391

[CIT0038] United Nations Department of Economic and Social Affairs, Population Division. (2020). *World population ageing 2020 highlights: Living arrangements of older persons (ST/ESA/SER.A/451)*. https://www.un.org/development/desa/pd/sites/www.un.org.development.desa.pd/files/undesa_pd-2020_world_population_ageing_highlights.pdf

[CIT0039] United Nations. (2017). *World population projected to reach 9.8 billion in 2050, and 11.2 billion in 2100*. https://www.un.org/development/desa/en/news/population/world-population-prospects-2017.html

[CIT0040] United Nations. (2019). *World population ageing 2019 highlights*. https://www.un.org/en/development/desa/population/publications/pdf/ageing/WorldPopulationAgeing2019-Highlights.pdf

[CIT0041] Webber, S. C., Porter, M. M., & Menec, V. H. (2010). Mobility in older adults: A comprehensive framework. Gerontologist, 50(4), 443–450. doi:10.1093/geront/gnq013 20145017

[CIT0042] World Health Organization. (2002). Active ageing: A policy framework. WHO. https://apps.who.int/iris/handle/10665/67215

[CIT0043] World Population Review. (2021). *Nigeria population 2021 (live)*. https://worldpopulationreview.com/countries/nigeria-population

[CIT0044] Ypofanti, M., Zisi, V., Zourbanos, N., Mouchtouri, B., Tzanne, P., Theodorakis, Y., & Lyrakos, G. (2015). Psychometric properties of the international personality item pool Big-Five personality questionnaire for the Greek population. Health Psychology Research, 3(2), 2206. doi:10.4081/hpr.2015.2206 26973962 PMC4768534

