# Supplementary material for: Testing the Webber’s Comprehensive Mobility Framework Using Self-Reported and Performance-Based Mobility Outcomes Among Community-Dwelling Older Adults in Nigeria
Source: Innov Aging. 2023 Mar 1;7(3):igad019. doi: 10.1093/geroni/igad019 (PMC10195698; doi:10.1093/geroni/igad019)
Supplement: igad019_suppl_Supplementary_Material [file igad019_suppl_supplementary_material.docx]

*Innovation in Aging* Online Supplementary Material. Ernest C. Nwachuwku, Daniel Rayner, Michael C. Ibekaku, Ekezie C. Uduonu, Charles I. Ezema, & Michael E. Kalu. Testing the Webber’s Comprehensive Mobility Framework using self-reported and performance-based mobility outcomes among community-dwelling older adults in Nigeria.

**Supplementary Table 1.** *Bivariate correlations of study variables in Wave 1*

| **Variable** | **1** | **2** | **3** | **4** | **5** | **6** | **7** | **8** | **9** | **10** | **11** | **12** | **13** | **14** | **15** | **16** | **17** | **18** |
| --- | --- | --- | --- | --- | --- | --- | --- | --- | --- | --- | --- | --- | --- | --- | --- | --- | --- | --- |
| 1. Age | - |  |  |  |  |  |  |  |  |  |  |  |  |  |  |  |  |  |
| 2. Sex | -0.29* | - |  |  |  |  |  |  |  |  |  |  |  |  |  |  |  |  |
| 3. Income | 0.03 | -0.32* | - |  |  |  |  |  |  |  |  |  |  |  |  |  |  |  |
| 4. Living arrangement | 0.10 | -0.07 | 0.05 | - |  |  |  |  |  |  |  |  |  |  |  |  |  |  |
| 5. Comorbidities ^a^ | 0.11 | 0.03 | -0.14 | 0.14 | - |  |  |  |  |  |  |  |  |  |  |  |  |  |
| 6. MoCA | -0.30* | -0.28* | 0.30* | 0.18 | -0.02 | - |  |  |  |  |  |  |  |  |  |  |  |  |
| 7. BMI | -0.01 | 0.05 | 0.19 | 0.01 | -0.06 | 0.02 | - |  |  |  |  |  |  |  |  |  |  |  |
| 8. MAP | -0.05 | -0.05 | 0.20 | -0.18 | 0.06 | -0.11 | 0.35* | - |  |  |  |  |  |  |  |  |  |  |
| 9. LEFS | -0.31* | 0.00 | 0.07 | -0.30* | -0.11 | 0.10 | -0.07 | 0.00 | - |  |  |  |  |  |  |  |  |  |
| 10. Occupation | 0.18 | 0.27* | -0.40* | 0.03 | -0.06 | -0.41* | 0.00 | -0.03 | -0.06 | - |  |  |  |  |  |  |  |  |
| 11. Education | -0.25* | -0.13 | 0.48* | 0.07 | 0.07 | 0.60* | 0.17 | -0.01 | 0.14 | -0.42* | - |  |  |  |  |  |  |  |
| 12. Marital status | 0.01 | 0.30* | -0.26 | 0.00 | 0.29* | -0.14 | -0.05 | 0.02 | -0.22* | 0.16 | -0.20 | - |  |  |  |  |  |  |
| 13. Exercise ^b^ | -0.08 | -0.05 | -0.15 | 0.03 | -0.01 | -0.10 | -0.17 | -0.17 | -0.08 | 0.01 | -0.05 | 0.13 | - |  |  |  |  |  |
| 14. Balance ^#^ | -0.41* | 0.14 | -0.21 | -0.31* | -0.02 | 0.16 | 0.08 | 0.01 | 0.34* | 0.03 | 0.09 | 0.05 | -0.06 | - |  |  |  |  |
| 15. Gait speed ^#^ | -0.42* | 0.10 | 0.06 | -0.25* | -0.03 | 0.15 | 0.10 | -0.06 | 0.43* | -0.07 | 0.20 | -0.11 | 0.00 | 0.58* | - |  |  |  |
| 16. Chair stands ^#^ | -0.36* | 0.10 | 0.06 | -0.15 | 0.00 | 0.33* | -0.02 | -0.01 | 0.24* | -0.11 | 0.27* | 0.04 | -0.02 | 0.39* | 0.41* | - |  |  |
| 17. Manty walk 2km | 0.20 | 0.09 | 0.00 | 0.11 | 0.09 | -0.21 | 0.11 | 0.05 | -0.53* | 0.15 | -0.17 | 0.01 | 0.12 | -0.20 | -0.25* | -0.22* | - |  |
| 18. Manty walk 0.5km | 0.28* | 0.03 | 0.10 | 0.11 | 0.18 | -0.11 | 0.03 | -0.03 | -0.64* | 0.09 | -0.12 | 0.09 | 0.09 | -0.26* | -0.26* | -0.27* | 0.82* | - |
| 19. Manty stair climb | 0.08 | 0.36* | -0.05 | 0.09 | 0.14 | -0.16 | 0.03 | -0.10 | -0.33* | 0.03 | 0.05 | 0.03 | -0.11 | -0.16 | -0.17 | -0.19 | 0.43* | 0.48* |

*Notes.* BMI = Body Mass Index; LEFS = Lower Extremity Functional Scale; MAP = Mean Arterial Pressure; MoCA = Montreal Cognitive Assessment

^a^ Number of comorbidities

^b^ Frequency of exercise (Self report)

^#^ Scores from balance, gait speed, and lower limb strength components of the Short Physical Performance Battery

* p-value < 0.05 (two-tailed)

**Supplementary Table 2.** *Bivariate correlations of study variables in Wave 2*

| **Variable** | **1** | **2** | **3** | **4** | **5** | **6** | **7** | **8** | **9** | **10** | **11** | **12** | **13** | **14** | **15** | **16** | **17** | **18** | **19** | **20** | **21** | **22** |
| --- | --- | --- | --- | --- | --- | --- | --- | --- | --- | --- | --- | --- | --- | --- | --- | --- | --- | --- | --- | --- | --- | --- |
| 1. Age | - |  |  |  |  |  |  |  |  |  |  |  |  |  |  |  |  |  |  |  |  |  |
| 2. Sex | 0.11 | - |  |  |  |  |  |  |  |  |  |  |  |  |  |  |  |  |  |  |  |  |
| 3. Income | -0.36* | -0.39* | - |  |  |  |  |  |  |  |  |  |  |  |  |  |  |  |  |  |  |  |
| 4. Living arrangement | -0.12 | -0.10 | 0.23* | - |  |  |  |  |  |  |  |  |  |  |  |  |  |  |  |  |  |  |
| 5. Comorbidities ^a^ | -0.03 | 0.27* | -0.43* | -0.13 | - |  |  |  |  |  |  |  |  |  |  |  |  |  |  |  |  |  |
| 6. MoCA | -0.14 | -0.35* | 0.30* | 0.14 | -0.21* | - |  |  |  |  |  |  |  |  |  |  |  |  |  |  |  |  |
| 7. BMI | 0.01 | 0.21* | 0.14 | 0.13 | -0.05 | 0.24* | - |  |  |  |  |  |  |  |  |  |  |  |  |  |  |  |
| 8. MAP | 0.09 | 0.08 | -0.17 | 0.00 | 0.20* | -0.08 | 0.22* | - |  |  |  |  |  |  |  |  |  |  |  |  |  |  |
| 9. Extraversion | 0.00 | -0.16 | -0.02 | 0.07 | 0.09 | -0.03 | -0.04 | -0.08 | - |  |  |  |  |  |  |  |  |  |  |  |  |  |
| 10. Agreeableness | 0.05 | -0.13 | -0.01 | 0.06 | -0.06 | 0.10 | 0.15 | -0.07 | 0.11 | - |  |  |  |  |  |  |  |  |  |  |  |  |
| 11. Conscientiousness | -0.18* | -0.27* | 0.12 | 0.15 | -0.12 | 0.27* | 0.15 | -0.11 | 0.03 | 0.44* | - |  |  |  |  |  |  |  |  |  |  |  |
| 12. Neuroticism | 0.03 | 0.03 | 0.23* | -0.11 | -0.21* | 0.11 | 0.23* | 0.02 | -0.07 | 0.24* | 0.20* | - |  |  |  |  |  |  |  |  |  |  |
| 13. Openness | -0.16 | -0.42* | 0.37* | 0.05 | -0.28* | 0.25* | 0.00 | -0.03 | 0.26* | 0.25* | 0.34* | -0.02 | - |  |  |  |  |  |  |  |  |  |
| 14. Occupation | 0.26* | 0.43* | -0.36* | -0.11 | 0.22* | -0.27* | 0.00 | 0.15 | -0.03 | -0.14 | -0.14 | 0.00 | -0.35* | - |  |  |  |  |  |  |  |  |
| 15. Education | -0.29* | -0.38* | 0.57* | 0.26* | -0.37* | 0.47* | 0.20* | -0.08 | -0.02 | 0.08 | 0.33* | 0.08 | 0.43* | -0.43* | - |  |  |  |  |  |  |  |
| 16. Marital status | 0.18* | 0.54* | -0.36* | -0.19* | 0.38* | -0.35* | 0.00 | 0.10 | -0.03 | -0.05 | -0.20* | 0.09 | -0.33* | 0.43* | -0.50* | - |  |  |  |  |  |  |
| 17. Exercise ^b^ | -0.06 | 0.02 | 0.04 | 0.02 | -0.08 | 0.04 | 0.11 | -0.01 | 0.09 | 0.19* | 0.10 | 0.09 | 0.08 | 0.02 | -0.01 | -0.05 | - |  |  |  |  |  |
| 18. Balance ^#^ | -0.23* | -0.17* | 0.13 | 0.22* | -0.34* | 0.19* | 0.01 | -0.26* | 0.02 | -0.02 | 0.12 | -0.02 | 0.08 | -0.19* | 0.16 | -0.19* | 0.01 | - |  |  |  |  |
| 19. Gait speed ^#^ | -0.35* | -0.27* | 0.49* | 0.21* | -0.44* | 0.24* | 0.18* | -0.22* | -0.01 | 0.06 | 0.27* | 0.11 | 0.14 | -0.29* | 0.53* | -0.36* | -0.05 | 0.26* | - |  |  |  |
| 20. Chair stands ^#^ | -0.30* | -0.29* | 0.21 | 0.09 | -0.21* | 0.09 | -0.10 | -0.16 | 0.19* | 0.04 | 0.29* | -0.18* | 0.34* | -0.30* | 0.28* | -0.36* | 0.02 | 0.28* | 0.30* | - |  |  |
| 21. Manty walk 2km | 0.02 | 0.34* | -0.17 | 0.05 | 0.24* | -0.06 | 0.14 | -0.09 | -0.01 | -0.19 | -0.28* | -0.07 | -0.08 | 0.14 | -0.20 | 0.20 | -0.07 | -0.17 | -0.20 | -0.30* | - |  |
| 22. Manty walk 0.5km | -0.01 | 0.26* | -0.16 | 0.09 | 0.23* | -0.10 | 0.12 | -0.08 | -0.02 | -0.16 | -0.21 | -0.08 | -0.04 | 0.20 | -0.17 | 0.16 | 0.00 | -0.06 | -0.14 | -0.27* | 0.89* | - |
| 23. Manty stair climb | -0.01 | 0.32* | -0.24 | -0.04 | 0.34* | -0.10 | 0.08 | -0.01 | -0.08 | -0.13 | -0.31* | -0.12 | -0.16 | 0.04 | -0.22* | 0.18 | -0.05 | -0.25* | -0.19 | -0.26* | 0.65* | 0.61* |

*Notes*. BMI = Body Mass Index; MAP = Mean Arterial Pressure; MoCA = Montreal Cognitive Assessment

^a^ Number of comorbidities

^b^ Frequency of exercise (Self report)

^#^ Scores from balance, gait speed, and lower limb strength components of the Short Physical Performance Battery

* p-value < 0.05 (two-tailed)

**Supplementary Table 3.** *Significant predictors of community dwelling older adults’ gait speed*

| **Variable** | **Wave 1** | | | **Wave 2** | | |
| --- | --- | --- | --- | --- | --- | --- |
|  | **B (95%CI)** | **β** | **p-value** | **B (95%CI)** | **β** | **p-value** |
| LEFS (Walk 1 Mile) | .325 (.084; .566) | .395 | .010 | - | - | - |
| Age | - | - | - | -.051 (-.081; -.021) | -.280 | .001 |
| No. of comorbidities | - | - | - | -.276 (-.421; -.131) | -.324 | .000 |
| BMI | - | - | - | .031 (.002; .061) | .175 | .036 |
| Openness | - | - | - | -.043 (-.077; -.009) | -.226 | .015 |
| Education (Secondary) | - | - | - | .731 (.199; 1.262) | .360 | .008 |
| Education (Tertiary) | - | - | - | .641 (.016; 1.266) | .279 | .045 |
| Adjusted R^2^ | .207 | | | .424 | | |

*Notes.* BMI = Body Mass Index; LEFS = Lower Extremity Functional Scale; MAP = Mean Arterial Pressure; MoCA = Montreal Cognitive Assessment scale; B = unstandardized beta; β = standardized beta. All predictors were entered into the model. For Wave 1, this included: age, sex, income, occupation, education, marital status, exercise, living arrangement, number of comorbidities, MoCA score, BMI, MAP, and modified LEFS. For Wave 2, this included: age, sex, income, occupation, education, marital status, exercise, living arrangement, number of comorbidities, MoCA score, BMI, MAP, extraversion, agreeableness, conscientiousness, neuroticism, and openness. Significance threshold was set at p-value < 0.05.

**Supplementary Table 4.** *Significant predictors of community dwelling older adults’ balance*

| **Variable** | **Wave 1** | | | **Wave 2** | | |
| --- | --- | --- | --- | --- | --- | --- |
|  | **B (95%CI)** | **β** | **p-value** | **B (95%CI)** | **β** | **p-value** |
| LEFS (Walk 1 Mile) | .283 (.077; .490) | .398 | .008 | - | - | - |
| Age | - | - | - | -.038 (-.064; -.012) | -.286 | .004 |
| Living arrangement | - | - | - | .046 (.001; .092) | .171 | .047 |
| No. of comorbidities | - | - | - | -.226 (-.352; -.099) | -.362 | .001 |
| Education (Primary) | - | - | - | -.506 (-.918; -.095) | -.337 | .016 |
| Education (Secondary) | - | - | - | -.520 (-.981; -.059) | -.359 | .028 |
| Adjusted R^2^ | .235 | | | .198 | | |

*Notes.* BMI = Body Mass Index; LEFS = Lower Extremity Functional Scale; MAP = Mean Arterial Pressure; MoCA = Montreal Cognitive Assessment scale; B = unstandardized beta; β = standardized beta. All predictors were entered into the model. For Wave 1, this included: age, sex, income, occupation, education, marital status, exercise, living arrangement, number of comorbidities, MoCA score, BMI, MAP, and modified LEFS. For Wave 2, this included: age, sex, income, occupation, education, marital status, exercise, living arrangement, number of comorbidities, MoCA score, BMI, MAP, extraversion, agreeableness, conscientiousness, neuroticism, and openness. Significance threshold was set at p-value < 0.05.

**Supplementary Table 5.** *Significant predictors of community dwelling older adults’ chair stands*

| **Variable** | **Wave 1** | | | **Wave 2** | | |
| --- | --- | --- | --- | --- | --- | --- |
|  | **B (95%CI)** | **β** | **p-value** | **B (95%CI)** | **β** | **p-value** |
| Age | - | - | - | -.044 (-.082; -.006) | -.209 | .025 |
| Extraversion | - | - | - | .029 (.001; .056) | .177 | .043 |
| Conscientiousness | - | - | - | .061 (.014; .108) | .242 | .011 |
| Neuroticism | - | - | - | -.038 (-.073; -.003) | -.193 | .034 |
| Adjusted R^2^ | .135 | | | .240 | | |

*Notes*. BMI = Body Mass Index; LEFS = Lower Extremity Functional Scale; MAP = Mean Arterial Pressure; MoCA = Montreal Cognitive Assessment scale; B = unstandardized beta; β = standardized beta. All predictors were entered into the model. For Wave 1, this included: age, sex, income, occupation, education, marital status, exercise, living arrangement, number of comorbidities, MoCA score, BMI, MAP, and modified LEFS. For Wave 2, this included: age, sex, income, occupation, education, marital status, exercise, living arrangement, number of comorbidities, MoCA score, BMI, MAP, extraversion, agreeableness, conscientiousness, neuroticism, and openness. Significance threshold was set at p-value < 0.05.

**Supplementary Table 6.** *Significant predictors of community dwelling older adults’ mobility limitation in walking 2 km*

| **Variable** | **Wave 1** | | **Wave 2** | |
| --- | --- | --- | --- | --- |
|  | **B (95%CI)** | **p-value** | **B (95%CI)** | **p-value** |
| LEFS (Walk 1 Mile) | -3.626 (-5.76; -1.492) | .001 | - | - |
| Marital Status (Widowed / Divorced / Single) | -2.995 (-5.878; -.113) | .042 | - | - |
| Female Sex | - | - | 3.986 (.528; 7.444) | .024 |
| No. of comorbidities | - | - | .981 (.200; 1.762) | .014 |
| Conscientiousness | - | - | -.318 (-.582; -.055) | .018 |
| Pseudo R^2^ | .536 | | .416 | |

Notes: BMI = Body Mass Index; LEFS = Lower Extremity Functional Scale; MAP = Mean Arterial Pressure; MoCA = Montreal Cognitive Assessment scale; B = unstandardized beta; β = standardized beta. All predictors were entered into the model. For Wave 1, this included: age, sex, income, occupation, education, marital status, exercise, living arrangement, number of comorbidities, MoCA score, BMI, MAP, and modified LEFS. For Wave 2, this included: age, sex, income, occupation, education, marital status, exercise, living arrangement, number of comorbidities, MoCA score, BMI, MAP, extraversion, agreeableness, conscientiousness, neuroticism, and openness. Significance threshold was set at p-value < 0.05.

**Supplementary Table 7.** *Significant predictors of community dwelling older adults’ mobility limitation in walking 0.5 km*

| **Variable** | **Wave 1** | | **Wave 2** | |
| --- | --- | --- | --- | --- |
|  | **B (95%CI)** | **p-value** | **B (95%CI)** | **p-value** |
| Living arrangement | -.411 (-.813; -.009) | .045 | - | - |
| LEFS (Walk 1 Mile) | -4.060 (-6.379; -1.742) | .001 | - | - |
| No. of comorbidities | - | - | 1.510 (.293; 2.727) | .015 |
| MAP | - | - | -.168 (-.319; -.016) | .030 |
| Conscientiousness | - | - | -.379 (-.713; -.045) | .026 |
| Pseudo R^2^ | .595 | | .419 | |

Notes: BMI = Body Mass Index; LEFS = Lower Extremity Functional Scale; MAP = Mean Arterial Pressure; MoCA = Montreal Cognitive Assessment scale; B = unstandardized beta; β = standardized beta. All predictors were entered into the model. For Wave 1, this included: age, sex, income, occupation, education, marital status, exercise, living arrangement, number of comorbidities, MoCA score, BMI, MAP, and modified LEFS. For Wave 2, this included: age, sex, income, occupation, education, marital status, exercise, living arrangement, number of comorbidities, MoCA score, BMI, MAP, extraversion, agreeableness, conscientiousness, neuroticism, and openness. Significance threshold was set at p-value < 0.05.

**Supplementary Table 8.** *Significant predictors of community dwelling older adults’ mobility limitation in stair climbing*

| **Variable** | **Wave 1** | | **Wave 2** | |
| --- | --- | --- | --- | --- |
|  | **B (95%CI)** | **p-value** | **B (95%CI)** | **p-value** |
| LEFS (Walk 1 Mile) | -1.593 (-2.873; -.314) | .015 | - | - |
| Female Sex | 4.467 (1.392; 7.542) | .004 | 2.972 (.338; 5.607) | .027 |
| No. of comorbidities | - | - | 1.012 (.305; 1.719) | .005 |
| Conscientiousness | - | - | -.276 (-.514; -.039) | .023 |
| Pseudo R^2^ | .433 | | .369 | |

Notes: BMI = Body Mass Index; LEFS = Lower Extremity Functional Scale; MAP = Mean Arterial Pressure; MoCA = Montreal Cognitive Assessment scale; B = unstandardized beta; β = standardized beta. All predictors were entered into the model. For Wave 1, this included: age, sex, income, occupation, education, marital status, exercise, living arrangement, number of comorbidities, MoCA score, BMI, MAP, and modified LEFS. For Wave 2, this included: age, sex, income, occupation, education, marital status, exercise, living arrangement, number of comorbidities, MoCA score, BMI, MAP, extraversion, agreeableness, conscientiousness, neuroticism, and openness. Significance threshold was set at p-value < 0.05.
